# A multicenter comparative study of the performance of four rapid immunochromatographic tests for the detection of anti-*Trypanosoma cruzi* antibodies in Brazil

**DOI:** 10.3389/fmed.2023.1031455

**Published:** 2023-03-02

**Authors:** Jacqueline Araújo Domingos Iturra, Leonardo Maia Leony, Fernanda Alvarenga Cardoso Medeiros, Job Alves de Souza Filho, Liliane da Rocha Siriano, Suelene Brito Tavares, Alejandro Ostermayer Luquetti, Vinícius Silva Belo, Andréa Silvestre de Sousa, Fred Luciano Neves Santos

**Affiliations:** ^1^Parasitic Diseases Service, Ezequiel Dias Foundation (FUNED), Belo Horizonte, Minas Gerais, Brazil; ^2^Advanced Public Health Laboratory, Gonçalo Moniz Institute, Oswaldo Cruz Foundation (FIOCRUZ-BA), Salvador, Bahia, Brazil; ^3^Chagas Disease Study Center (NEDoC), University Hospital, Federal University of Goiás (UFG), Goiânia, Goiás, Brazil; ^4^Department of Health Sciences, Federal University of São João Del-Rei (UFSJ), Divinópolis, Minas Gerais, Brazil; ^5^Evandro Chagas National Institute of Infectious Diseases, Oswaldo Cruz Foundation (FIOCRUZ-RJ), Rio de Janeiro, Brazil; ^6^Integrated Translational Program in Chagas Disease from FIOCRUZ (Fio-Chagas), Oswaldo Cruz Foundation (FIOCRUZ-RJ), Rio de Janeiro, Brazil

**Keywords:** Chagas disease, serology, rapid diagnostic tests, performance, screening

## Abstract

Diagnosis of *Trypanosoma cruzi* (*T. cruzi*) infection in the chronic phase of Chagas disease (CD) is performed by serologic testing. Conventional tests are currently used with very good results but require time, laboratory infrastructure, and expertise. Rapid diagnostic tests (RDTs) are an alternative as the results are immediate and do not require specialized knowledge, making them suitable for epidemiologic studies and promising as a screening tool. Nevertheless, few studies conducted comparative evaluations of RDTs to validate the results and assess their performance. In this study, we analyzed four trades of rapid tests (OnSite Chagas Ab Combo Rapid Test-United States, SD Bioline Chagas AB-United States, WL Check Chagas-Argentina, and TR Chagas Bio-Manguinhos-Brazil) using a panel of 190 samples, including sera from 111 infected individuals, most of whom had low *T. cruzi* antibody levels. An additional 59 samples from uninfected individuals and 20 sera from individuals with other diseases, mainly visceral leishmaniasis, were included. All tests were performed by three independent laboratories in a blinded manner. Results showed differences in sensitivity from 92.8 to 100%, specificity from 78.5 to 92.4%, and accuracy from 90.5 to 95.3% among the four assays. The results presented here show that all four RDTs have high overall diagnostic ability. However, WL Check Chagas and TR Chagas Bio-Manguinhos were considered most suitable for use in screening studies due to their high sensitivity combined with good performance. Although these two RDTs have high sensitivity, a positive result should be confirmed with other tests to confirm or rule out reactivity/positivity, especially considering possible cross-reactivity with individuals with leishmaniasis or toxoplasmosis.

## 1. Introduction

Chagas disease (CD) is a life-threatening, neglected tropical disease caused by the hemoflagellate *Trypanosoma cruzi* (*T. cruzi*). This parasite is responsible for an average of 12,000 deaths per year, and it is estimated that between 6 and 7 million people are infected worldwide (1, 2). However, despite the high mortality and morbidity, only 7% of *T. cruzi* carriers in Latin America are diagnosed and only about 1% receive etiologic treatment (3). *T. cruzi* is responsible for the highest parasitic disease burden in 21 Latin American countries, with a high prevalence in the southern Cone (4), where it is transmitted to humans mainly through contact with contaminated feces or urine from bloodsucking triatomine insects, also known as kissing bugs. Other routes of infection include congenital transmission, oral ingestion of contaminated food or beverages, transfusion of blood or blood products, and organ donation. Increasing international migration flows to non-endemic regions have led to the spread of *T. cruzi* infection beyond the borders of Latin America and have become a global health problem (5–7).

Successful diagnosis of CD depends on the stage of the disease, as different approaches (*in vitro* diagnostic (IVD) techniques) are used for each phase: an initial acute phase and a lifelong chronic phase. In the acute phase, which lasts up to two/three months, parasitological or molecular biology-based methods are typically used, while indirect serological methods such as indirect hemagglutination (IHA), enzyme-linked immunosorbent assay (ELISA), indirect immunofluorescence (IIF), chemiluminescence (CLIA), and electrochemiluminescence immunoassay (ECLIA) are used in the lifelong chronic phase (8). Although serological tests currently have high diagnostic performance, they require complex, specialized infrastructure and qualified personnel to perform. Therefore, IVD serological tests can be a significant barrier to access to diagnosis. The development of point-of-care (POC) devices such as rapid diagnostic tests (RDTs) has highlighted a way to circumvent the need for specialized infrastructure and personnel. These devices are designed to be simple, convenient, and intuitive to use. They require no refrigeration, no specialized infrastructure, no trained personnel, and no further processing by the user to obtain a result. Therefore, POC tests can be used to screen CD affected individuals, especially those living in rural or remote areas with limited access to health care. A negative RDT result excludes the disease, while positive results should be forwarded for diagnostic confirmation with other serological tests to exclude or confirm CD as recommended by the World Health Organization (WHO) (9, 10). Particular attention should be paid to the sensitivity of RDTs used as screening tests. A test with higher sensitivity (100%) is advisable because low sensitivity of the first level of testing in a screening algorithm may lead to excessive false-negative results and exclude people from accurate diagnosis, thereby underestimating the number of infected individuals. This strategy may improve access to diagnosis and treatment. Recently, the Pan American Health Organization (PAHO) recommended the use of ELISA or RDT as the sole test for seroepidemiologic testing (11).

Regarding the inconsistent diagnostic performance when using serological tests in different settings, some differences have been reported in the literature with respect to the parasite and the seven discrete typing units (DTUs) recognized today (12–14). However, other reports have found similar results when using conventional serology with samples from Mexico (mainly lineage TcI) (15) and also when using a single RDT with sera from different countries with lineages TcI, II and V, the main DTUs from endemic regions (16). In this study, samples from one region (Brazil) were used.

Considering the predicaments herein set forth, we endeavored to perform a multicenter systematic evaluation of the diagnostic performance of RDT kits available in Brazil. This is the first study comparing the performance of RDTs in Brazil for the diagnosis of chronic Chagas disease.

## 2. Materials and methods

### 2.1. Selection of RDTs

All commercial RDTs registered with the Brazilian Health Regulatory Agency (ANVISA) were included in this study. A total of four RDTs from four different manufacturers were available: OnSite Chagas Ab Combo Rapid Test® (CTK Biotech, United States), SD Bioline Chagas AB® (Abbott, USA), WL Check Chagas® (Wiener lab., Rosário, Argentina), and TR Chagas Bio-Manguinhos® (Bio-Manguinhos, Fiocruz, Rio de Janeiro, Brazil). The RDTs were sent by the General Coordination of Public Health Laboratories (CGLAB, Ministry of Health, Brazil) to each participating reference laboratory *via* a commercial shipping service. Importantly, RDTs from each brand were from the same batch.

### 2.2. Participating reference laboratories

The study was conducted in three participating Brazilian reference laboratories: The Advanced Public Health Laboratory (LASP) at the Gonçalo Moniz Institute (FIOCRUZ) in Salvador, Bahia; the Parasitic Diseases Service of the Ezequiel Dias Foundation (FUNED) in Belo Horizonte, Minas Gerais; and the Chagas Disease Study Center (NEDoC) at the Federal University of Goiás (UFG) in Goiânia, Goiás. All three participating reference laboratories performed the four RDTs with the same sample set. All participating laboratories adhered to Good Laboratory Practice and sample reactivity was repeated using conventional serology after the serum samples were thawed in the laboratory that provided the samples.

### 2.3. Sample collection

With an expected error of 2%, sensitivity of 99%, specificity of 99.5%, and confidence interval of 95%, the minimum sample number was 48 sera from negative individuals and 96 sera from *T. cruzi*-positive individuals. We included 59 sera from *T. cruzi*-negative and 111 sera from *T. cruzi*-positive individuals from the existing sera bank at NEDoC. The *T. cruzi*-positive samples were previously collected from individuals with the chronic phase of CD with known epidemiological and clinical data (usually heart disease and/or megacolon and/or megaesophagus). These infected and uninfected individuals were tested in the laboratory at the request of Goias State physicians to confirm or exclude the diagnosis. This sample group consisted predominantly of samples with low or moderate reactivity in the serological tests: titration of less than 1:640 in IIF and IHA; reactivity indices between 1.2 and 2.0 (low reactivity) and 2.1 to 3.0 (moderate reactivity) in conventional ELISA. In addition, positive sera for visceral leishmaniasis (VL; *n* = 10), mucocutaneous leishmaniasis (CL; *n* = 6), and toxoplasmosis (TOX; *n* = 4) from the FUNED serum bank were included to evaluate cross-reactivity. All samples were thawed at −20°C without additional preservatives and previously tested for *T. cruzi* infection: indirect immunofluorescence (IIF; Anti-human IgG conjugated to fluorescein, Biomerieux® Marcy L’Etoile), indirect hemagglutination (IHA; Chagatest HAI screening A-V®, Wiener lab, Rosario, Argentina), ELISA with crude antigens (Teste ELISA para Chagas III®, Grupo Bios, Santiago, Chile), recombinant ELISA (Chagatest ELISA, recombinant v.3.0®, Wiener lab, Rosario, Argentina), chemiluminescence microparticle immunoassay (CMIA; Architect Chagas, Abbott Laboratories, Abbott GmbH, Wiesbaden, Germany), enzyme-linked immunosorbent assay (ELISA) with lysate/recombinant antigens (Gold ELISA Chagas®, REM Industry and Commerce Ltd., São Paulo, Brazil). Samples were aliquoted and coded so that members of the participating reference laboratory teams had no knowledge of their reactivity. Serum aliquots stored in dry ice were shipped by CGLAB to each participating reference laboratory using a commercial shipping service. The serological results for each serum using each of the serological techniques are shown in a Supplementary Table S1.

### 2.4. Immunochromatographic assays

RDTs were performed according to the technical instructions of the respective manufacturer. In each participating reference laboratory, the same sera were evaluated for all four RDTs. The results were read by two independent observers from each participating institution. In cases of doubt or disagreement, a third observer was consulted and the tests were repeated if consensus could not be reached. Final results were sent to the serum bank supervisor, who was the only person who knew the serological profile of the samples. A test was considered invalid if the control line was missing. After completion of the laboratory analysis, a consensus result between the three participating reference laboratories was compared with the serological profile of the samples and the performance of each RDT was determined.

### 2.5. Usability assessment

The criterion of ease of use in performing RDTs was quantified and compared. At the end of the study, the technical staff responsible for conducting the tests for the study were asked to complete a usability questionnaire for each RDT. This questionnaire was adapted from a conventional format used in several similar studies led by WHO/Foundation for Innovative New Diagnostics (FIND)/Médecins Sans Frontières (MSF)/Epicenter in 2001 (17, 18) and also in another international study on RDTs (19). The questionnaire was used to distinguish and evaluate the general characteristics of the tests and to assess the perception of the technical staff regarding the ease of use in performing each test. The questionnaires included information on the number of invalid tests, shelf life, storage temperature, amount of blood/serum/plasma required, number of steps and time required to perform the test, stability of results, additional material required, ease of opening the package, ease of performing the test, ease of identifying reagents, quality of instructions for use, and cost. Each item of the questionnaire was assigned an individual score, with a higher score indicating a more positive response. A total of 26 items could be evaluated.

### 2.6. Statistical analysis

**Figure 1:**
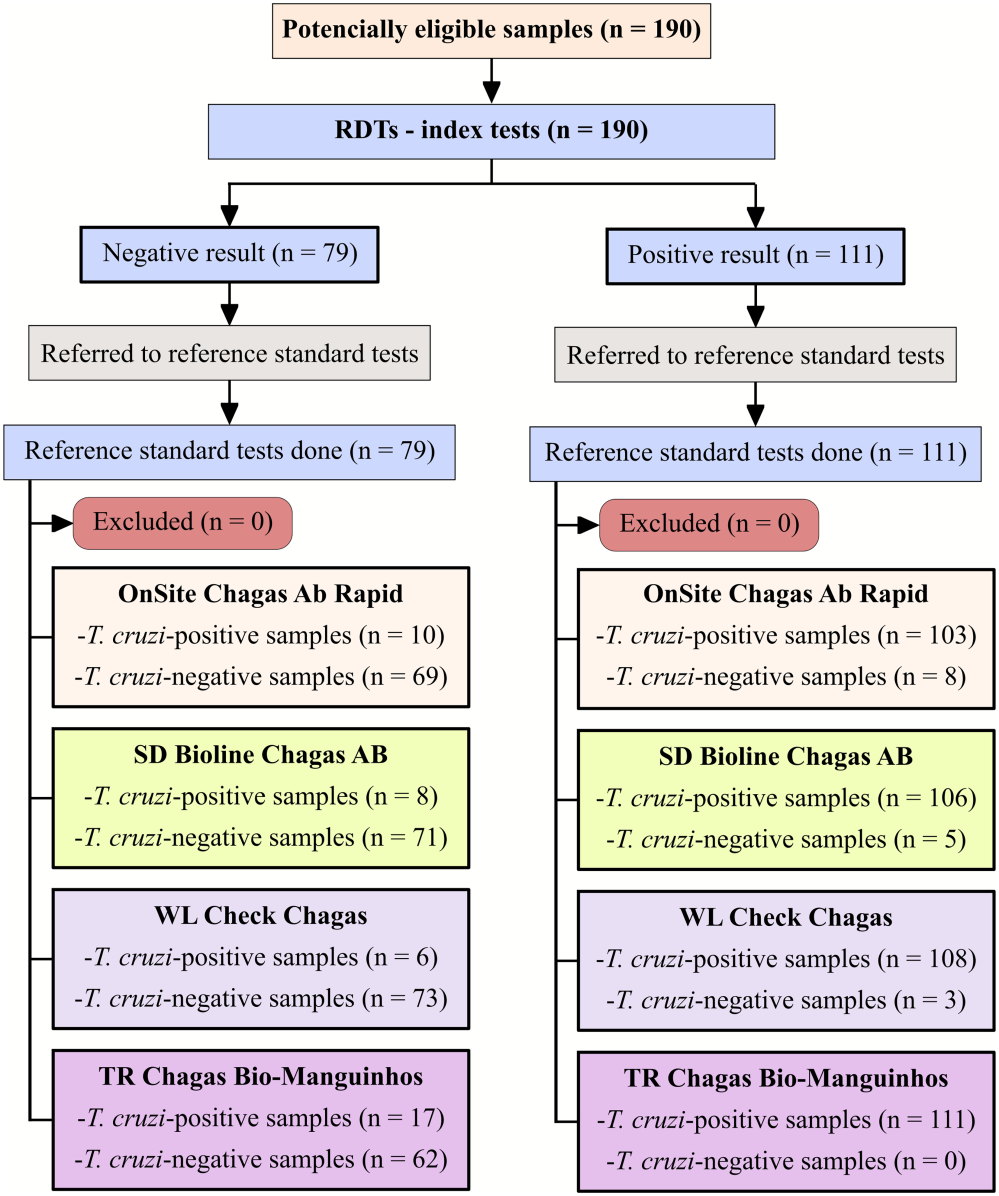
Flowchart depicting study design in accordance with the Standards for Reporting of Diagnostic Accuracy Studies (STARD) guidelines.

To obtain a robust assessment of the performance of each kit, statistical tools were used by calculating the following diagnostic test parameters: Sensitivity (the probability of a test being positive in the presence of infection), Specificity (the probability of a test being negative in the absence of infection), Accuracy (the ability of a test to discriminate between target disease and health status), and Predictive Values (20, 21) using a dichotomous approach (2 × 2 contingency table). Confidence intervals (CI) were determined at a 95% confidence level (95% CI), and the absence of overlapping 95% CI bars was used to infer statistical significance (22). Positive and negative predictive values were estimated for different prevalence scenarios. The chance of false-positive versus true-positive and false-negative versus true-negative results were calculated for the following prevalence values of chronic Chagas disease: 0.1, 1, 5, and 10%. The strength of agreement between the results of the RDTs and the serological profile of the samples was assessed using the Cohen’s *kappa* coefficient (*κ*) and interpreted as follows: poor (*κ* = 0), slight (0 < *κ* ≤ 0.20), fair (0.21 < *κ* ≤ 0.40), moderate (0.41 < *κ* ≤ 0.60), substantial (0.61 < *κ* ≤ 0.80), and almost perfect (0.81 < *κ* ≤ 1.0) agreement (23). Performance parameters were obtained using MedCalc for Windows v. 20.190 (MedCalc Software, Ostend, Belgium), whereas graphs were generated using GraphPad Prism 9 graphing software (San Diego, CA, United States). A study flowchart (Figure 1) and checklist were prepared according to STARD guidelines (24).

## 3. Results

A total of 190 serum samples were tested with four IgG *T. cruzi* RDTs (Supplementary Table S1). IgG survey in serum samples from 111 *T. cruzi*-positive samples showed variable values of sensitivity, ranging from 92.8% for OnSite Chagas Ab Combo Rapid Test, 95.5% for SD Bioline Chagas AB, and 97.3% for WL Check Chagas to 100% for TR Chagas Bio-Manguinhos (Table 1). For *T. cruzi*-negative samples, the highest value of specificity was obtained with WL Check Chagas (92.4%). A lower value was observed for SD Bioline Chagas AB, OnSite Chagas Ab Combo Rapid Test, and TR Chagas Bio-Manguinhos, which had specificity values of 89.9, 87.3, and 78.5%, respectively. Accuracy reached the highest value when samples were tested with WL Check Chagas (95.3%). A lower value was observed for SD Bioline Chagas AB (93.2%), TR Chagas Bio-Manguinhos (91.1%) and OnSite Chagas Ab Combo Rapid Test (90.5%).

**Table 1:** Diagnostic performance and strength of agreement of four rapid diagnostic tests for the detection of *Trypanosoma cruzi* IgG.

Performance parameters	OnSite Chagas Ab combo rapid test	WL check Chagas	SD Bioline Chagas	TR Chagas Bio-Manguinhos
SEN % (95%CI)	92.8 (86.3–96.8)	97.3 (92.3–99.4)	95.5 (89.8–98.5)	100 (96.7–100)
SPE % (95%CI)	87.3 (78.0–93.8)	92.4 (84.2–97.2)	89.9 (81.0–95.5)	78.5 (68.2–86.1)
ACC % (95%CI)	90.5 (85.5–93.9)	95.3 (91.2–97.5)	93.2 (88.6–96.0)	91.1 (86.1–94.3)
*k* (95% CI)	0.80 (0.72–0.89)	0.90 (0.84–0.96)	0.86 (0.78–0.93)	0.81 (0.72–0.90)

SEN, sensitivity; SPE, specificity; ACC, accuracy; *k*, Cohen’s Kappa coefficient; CI, confidence interval.

Qualitative evaluation of the results using Cohen’s *Kappa* method showed substantial agreement between the OnSite Chagas Ab Combo Rapid Test and the reference tests. For all other RDTs, qualitative evaluation of the results showed almost perfect agreement with the reference tests. Considering the 95% CI overlap, sensitivity, specificity, accuracy, and Cohen’s *Kappa* index showed no differences among the four RDTs (Table 1).

**Figure 2:**
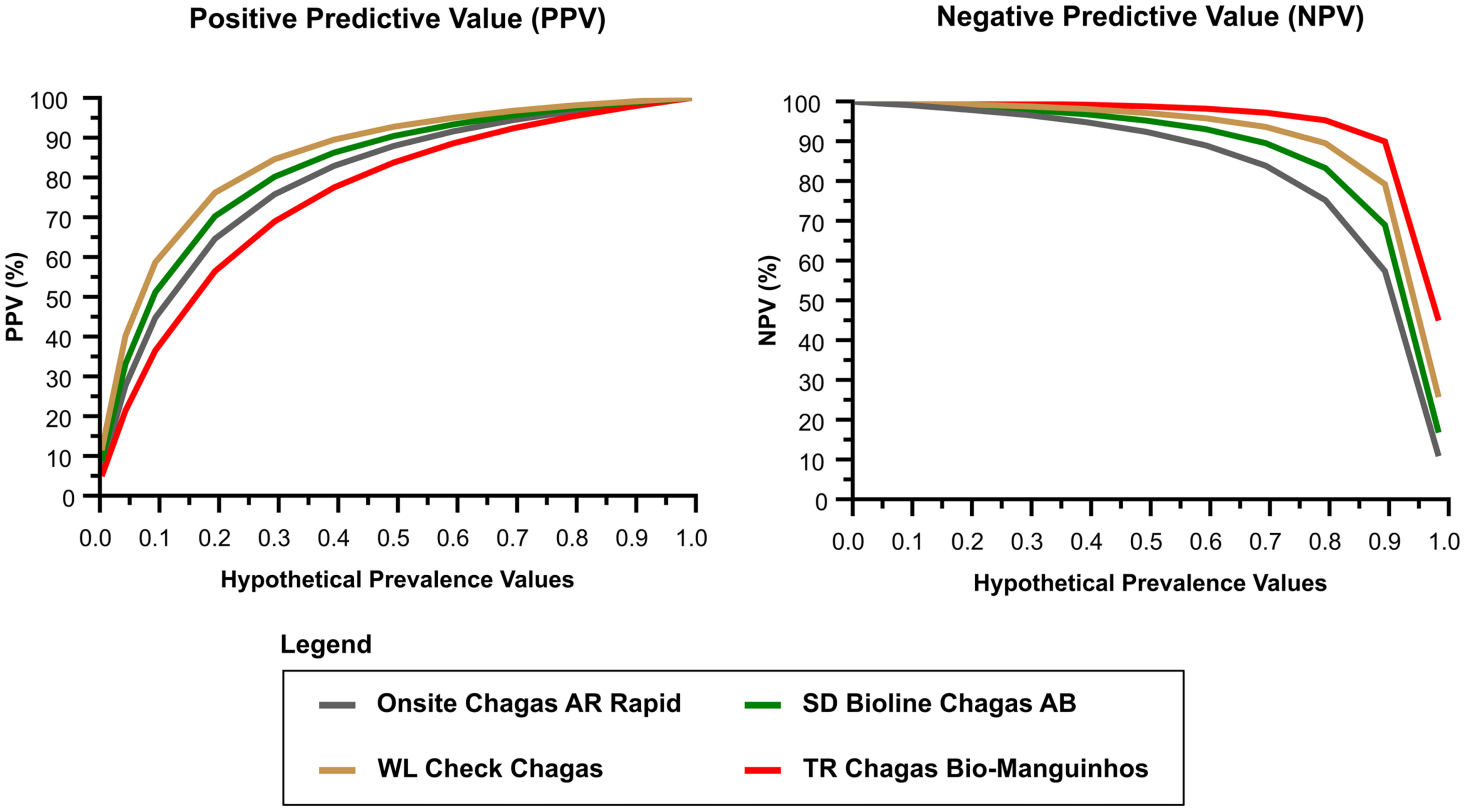
Positive and negative predictive estimates for different prevalence scenarios of chronic Chagas disease. NPV, negative predictive value; PPV, positive predictive value.

The positive and negative predictive values were also estimated. Because the true prevalence of chronic CD varies from region to region, we used a hypothetical prevalence range to evaluate different scenarios. Figure 2 summarizes the association between the predictive values and the hypothetical prevalence scenarios. Decreasing prevalence resulted in low positive predictive values for all RDTs. Regarding the ratio of false-positive/negative to true-positive/negative, hypothetical prevalence values were used to represent most scenarios in which testing is performed. The chance of false-negative results relative to true-negative results was low for all tests and prevalence values (Table 2). On the other hand, the chance of false-positive results was predominantly high for any true-positive result, especially for low prevalence values (0.1 and 1%).

**Table 2:** Chance of false positive in relation to true positives and of false negatives in relation to true negatives for different prevalence values of chronic Chagas disease.

Prevalence	OnSite Chagas Ab Combo rapid test	WL check Chagas	SD Bioline Chagas	TR Chagas Bio-Manguinhos
	False positives: true positives			
0.1%	136.4	78.3	105.1	214.8
1%	13.5	7.7	10.5	21.3
5%	2.6	1.5	2.0	4.1
10%	1.2	0.7	0.9	1.9
*False negative: true negatives*				
0.1%	<0.001	<0.001	<0.001	NS
1%	<0.001	<0.001	<0.001	NS
5%	0.004	0.002	0.003	NS
10%	0.009	0.003	0.006	NS

**Figure 3:**
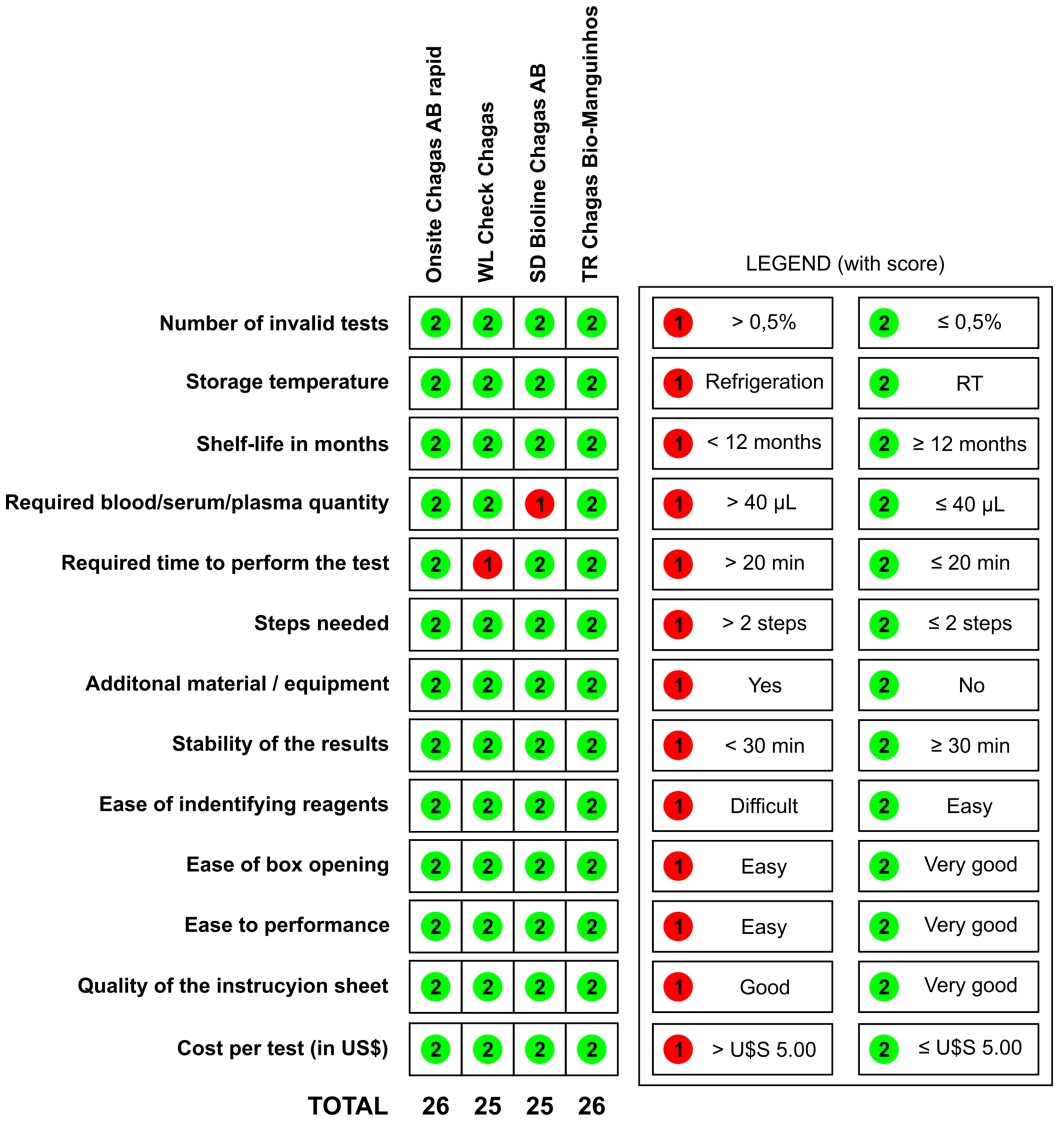
Validity and inter-reader reliability of four rapid diagnostic tests for the detection of IgG anti-*Trypanosoma cruzi*. RT (room temperature).

Regarding usability assessment, all RDTs were found to have the same storage conditions (room temperature ≤ 30°C), require the same biological sample (whole blood, plasma, or serum), and results are stable for up to 30 min. Invalid tests were reported for <0.5% of RDTs performed. For all four RDTs, ease of performance, ease of opening the package, and interpretation of results were described as “very easy.” The quality of the RDT instructions was described as “very good” for all RDTs. Some differences in the amounts of blood or serum/plasma required were noted for all four tests: OnSite Chagas Ab Combo Rapid Test and WL Check Chagas require 40 μl of blood, TR Chagas Bio-Manguinhos requires 10 μl, while SD Bioline Chagas AB requires 100 μl, the largest amount among them. None of the RTD assays require a device to read the results, so they can be used in field studies. OnSite Chagas Ab Combo Rapid Test, WL Check Chagas, SD Bioline Chagas AB and TR Chagas Bio-Manguinhos are one-step assays. As shown in Figure 3, TR Chagas Bio-Manguinhos and OnSite Chagas Ab Combo Rapid Test scored the highest (= 26), followed by SD Bioline Chagas AB and WL Check Chagas (score = 25).

## 4. Discussion

RDTs represent an interesting strategy for screening at-risk populations for acquisition of CD in low-resource and high-risk settings in endemic countries. WHO has set global targets and milestones for 2030 to eliminate transmission of *T. cruzi* through four modes of transmission (vectorial, transfusion, transplantation, and congenital) and achieve 75% coverage of the target population with antiparasitic treatment in 15 endemic countries in Latin America (25). This is an ambitious goal, as only 7% of *T. cruzi* carriers have been diagnosed and about 1% receive etiologic treatment (3). Thus, improving access to and demand for effective diagnosis, treatment, and care for CD is critical to controlling CD. Unfortunately, access to CD diagnostics remains one of the main barriers to control of this disease, as diagnosis in the chronic phase depends on laboratory infrastructure and qualified personnel. *In vitro* diagnostic tests at the point of care, such as RDTs, offer a promising strategy to address the gap in access to diagnosis that exists in many limited and isolated communities in endemic areas. However, similar to the enzyme-linked immunosorbent assay (ELISA), the performance of POC-IVD devices depends on the antigen preparation used, which warrants a systematic evaluation of their diagnostic performance (13). In this article, we evaluated the performance of four RTDs in the diagnosis of CD using samples from different Brazilian endemic areas.

In a comparative evaluation of 11 commercially available RDTs conducted by several national reference laboratories worldwide using a diverse panel of 474 samples, OnSite Chagas Ab Combo Rapid Test achieved a sensitivity of 90.1% and a specificity of 91%. In the same study, SD Chagas Ab Rapid showed a sensitivity of 90.7% and a specificity of 94% (19). Interestingly, WL Check Chagas showed a sensitivity of 88.7% and a specificity of 97%. Except for the sensitivity of WL Check Chagas, the sensitivity and specificity values of the other RDTs in the present study were within the 95% CI (19). According to the manufacturer, the sensitivity of WL Check Chagas was evaluated using four commercial serological panels with a total of 62 positive sera, and 61/62 (~98%) samples were correctly identified. However, the manufacturer reports a lower sensitivity (93.9%; 95% CI 91.1–96.6%) when the test was evaluated using a panel of 326 samples characterized by ELISA and IHA. Considering the confidence interval, both evaluations were consistent with the sensitivity observed in the present study (97.3%; 95% CI 92.3–99.4%). Similar sensitivity values were observed when serum samples were used during a WL Check Chagas field study (95.7%), although sensitivity was lower when the test examined whole blood (87.3%) (26). Accordingly, the manufacturer reported lower sensitivity (91.5%) when this RDT analyzed whole blood rather than plasma/serum. A possible interpretation for these differences in sensitivity is that different batches were used and the possibility exists that manufacturers changed the composition and proportions of the different antigens originally used after the results of the first reported studies (19) with lower sensitivity eight years ago. Information on which *T. cruzi* antigens were used in WL Check Chagas, SD Chagas Ab Rapid, and OnSite Chagas Ab Combo Rapid Test was not disclosed by the manufacturers.

In the present study, TR Chagas Bio-Manguinhos, using two recombinant *T. cruzi*-chimeric antigens deposited in different lines (27), correctly identified all positive samples and achieved a sensitivity of 100% (95% CI 96.7–100%) and a specificity of 78.5 (95% CI 68.2–86.1). This test is the most recent addition to the repertoire of available POC tests for CD, so there is a lack of independent studies evaluating its diagnostic performance. However, there are numerous studies evaluating the performance of these antigens in other IVD systems (27–35) and mammalian hosts (36–38). In a study of 280 CD-positive samples, IBMP-8.1 antigen showed a sensitivity of 98.9% (95% CI 96.9–99.6%) when used in an ELISA format and 98.6% (95% CI 96.4–99.4%) in a liquid microarray system, while IBMP-8.4 showed a sensitivity of 99.6% (95% CI 98–99.9%) in ELISA and 98.9% (95% CI 96.9–99.6%) in a microarray system (39). Similar results were obtained in a phase II study in Brazil (40), and the antigens maintained their performance in other studies in Argentina (41) and Spain (30). Moreover, no cross-reactions with visceral and mucocutaneous leishmaniasis were observed with IBMP-8.4 under ELISA or liquid microarray systems, while IBMP-8.1 in liquid microarray did not cross-react with visceral leishmaniasis, but cross-reactions for mucocutaneous leishmaniasis were observed in an IBMP-8.1 ELISA (0.7%) (32). Interestingly, the structural stability of IBMP chimeric antigens over time, pH and temperature variations, and in buffer systems was investigated. The structure and diagnostic performance were maintained under adverse conditions, suggesting a robust design (32). This robustness favors use in POC assays, as these devices must withstand harsh environments and be reliable enough to be easily used, interpreted, and stored.

The usability evaluation showed that no invalid result was obtained when *T. cruzi*-positive and negative samples were tested with all four RTDs. In terms of storage temperature, shelf life in months, stability of results, ease of reagent identification, ease of package opening, ease of performance, and quality of instructions, all four RTDs achieved similar results. OnSite Chagas Ab Combo Rapid Test, WL Check Chagas, and TR Chagas Bio-Manguinhos require volumes of up to 40 μl of whole blood, whereas SD Bioline Chagas AB requires 100 μl, a volume that is difficult to obtain by digital puncture, making this test unusable for epidemiological studies and as a screening tool. No RDT requires equipment to read results, making it feasible to use in the field. In addition, no assessed assay requires more than two steps to perform. For the WL Check Chagas, the test took more than 20 min to perform. WL Check Chagas and SD Bioline Chagas AB were the most complex tests (score = 25), while the highest score was achieved by TR Chagas Bio-Manguinhos and OnSite Chagas Ab Combo Rapid Test (score = 26). Of the four assays evaluated, the WL Check Chagas and TR Chagas Bio-Manguinhos were considered the most suitable for use in screening studies because they are reliable and highly sensitive for the diagnosis of CD. According to the instructions of all four kits evaluated, the test result is independent of the type of biological sample used for the immunoassay, whether blood, serum, or plasma.

The main limitation of this study was the restriction on the use of samples with low or moderate reactivity in the serological tests (titration of less than 1:640 for IIF and IHA; reactivity indices between 1.2 and 2.0 (low reactivity) and 2.1 to 3.0 (moderate reactivity) for conventional ELISA). The selection of samples with low or moderate reactivity may lead to a decrease in the sensitivity values of the evaluated RDTs, which may not correspond to their use in a real population. However, the predominance of samples with these characteristics was propositaly intended to detect infected individuals with low titers, as in conventional serology, and to avoid the possible loss of infected individuals. Another limitation concerns the lack of band intensity analysis. This would be particularly important to verify the intensity of false-positive bands. However, visual analysis revealed bands of varying intensity for false-positive lines. Despite a consistent detection pattern of the control lines, we observed that false-positive results exhibited whitish spots over the antigen reaction area, while others showed bright to almost faint colors as a positive sign of detection. The presence of these whitish spots or faint bands over the antigen reaction area led to an increase in the number of false-positive results in low CD prevalence scenarios. Indeed, at prevalence values of 0.1 and 1%, the chance of false-positive results was predominantly high for each true-positive result, whereas false-negative results were low relative to true-negative results for all tests and all prevalence values.

The results presented here show that all four RDTs have high overall diagnostic ability. We believe that the antigenic variability of *T. cruzi* did not affect the performance evaluation of the RDTs, since we used only Brazilian samples. Indeed, sera from individuals infected in Mexico (a region with TcI) have shown similar reactivity on conventional serology (15). Also, previous studies using other RDT (Chagas Stat-Pak), that was not included in this study because it does not have a current registration with ANVISA, performed with sera from different countries showed no differences in terms of different DTU (Tc I-II-V) in different regions of Latin America (16). Due to the overlap of 95% CI values, no differences were observed between the results for sensitivity, specificity, and accuracy. The high sensitivity values ensure that most (if not all) positive individuals are correctly diagnosed and referred to medical care. In the absence of laboratory facilities, the increased use of these rapid tests, which are reliable, cheap, and simple enough to be used by non-laboratory personnel, should contribute significantly to the effective control of CD and improve diagnosis and treatment, especially in remote and rural areas in endemic countries.

## Data availability statement

The original contributions presented in the study are included in the article/Supplementary material, further inquiries can be directed to the corresponding author.

## Ethics statement

The studies involving human participants were reviewed and approved by Institutional Review Board (IRB) for Human Research of the Gonçalo Moniz Institute (CAAE 67809417.0.0000.0040), the Ezequiel Dias Foundation (CAAE 21538619.4.2002.9507), and the Federal University of Goiás (CAAE 21538619.4.2001.5078). The ethics committee waived the requirement of written informed consent for participation.

## Author contributions

AL and FS designed the experimental procedure. JI, LL, FM, JF, and LS performed the RDT assays. VB and FS performed the statistical analysis. LL and FS wrote the article. JI, FM, JF, LS, ST, AL, VB, and AS helped to write the article. JI, LL, FM, JF, LS, ST, and FS performed data collection, analysis, and interpretation. AL provided the biological samples. FS prepared the illustrations and supervised the work. JI, AL, and FS provided the laboratory space. AS and FS obtained funding for this study. All authors contributed to the article and approved the submitted version.

## Funding

This research was supported by the Coordination for the Improvement of Higher Education Personnel in Brazil (CAPES; Finance Code 001 award to LL and FS). FS is a research grantee of the National Council for Scientific and Technological Development-Brazil (CNPq; grant number 309263/2020–4). The funders had no influence on the study design, data collection and analysis, decision to publish, or preparation of the manuscript.

## Conflict of interest

AS and FS are employees of FIOCRUZ and one of the RDTs was produced by a subsidiary of FIOCRUZ (Bio-Manguinhos), but they are not involved in the production of this kit (TR Chagas Bio-Manguinhos).

The remaining authors declare that the research was conducted in the absence of any commercial or financial relationships that could be construed as a potential conflict of interest.

## Publisher’s note

All claims expressed in this article are solely those of the authors and do not necessarily represent those of their affiliated organizations, or those of the publisher, the editors and the reviewers. Any product that may be evaluated in this article, or claim that may be made by its manufacturer, is not guaranteed or endorsed by the publisher.
