# Supplementary material for: A multicenter comparative study of the performance of four rapid immunochromatographic tests for the detection of anti-Trypanosoma cruzi antibodies in Brazil
Source: Front Med (Lausanne). 2023 Mar 2;10:1031455. doi: 10.3389/fmed.2023.1031455 (PMC10017777; doi:10.3389/fmed.2023.1031455)
Supplement: Supplementary file 1 [file Table_1.pdf]

Supplementary Table 1

Reference standard tests and index tests results

Sample ID	Reference standard tests results			Final result	Index tests results (Rapid Diagnostic Tests - RDTs)			
	EIA	IIF	IHA		Onsite Chagas	WL Check Chagas	SD Bioline Chagas Ab	TR Chagas Bio-Manguinhos
34615	2.1	160	40	***T. cruzi*-positive**	Positive	Positive	Positive	Positive
9663	2.5	320	80	***T. cruzi*-positive**	Positive	Positive	Positive	Positive
9939	2.7	80	80	***T. cruzi*-positive**	Positive	Positive	Negative	Positive
11375	3.2	160	80	***T. cruzi*-positive**	Positive	Positive	Positive	Positive
11434	1.6	160	80	***T. cruzi*-positive**	Positive	Positive	Positive	Positive
14755	4.1	320	80	***T. cruzi*-positive**	Positive	Positive	Positive	Positive
16050	1.4	320	80	***T. cruzi*-positive**	Positive	Positive	Positive	Positive
18557	3.1	320	80	***T. cruzi*-positive**	Positive	Positive	Positive	Positive
18803	2.3	320	80	***T. cruzi*-positive**	Positive	Positive	Positive	Positive
18980	1.8	160	80	***T. cruzi*-positive**	Positive	Positive	Positive	Positive
21560	1.6	320	80	***T. cruzi*-positive**	Positive	Positive	Positive	Positive
31251	3.1	640	80	***T. cruzi*-positive**	Positive	Positive	Positive	Positive
32632	1.3	160	80	***T. cruzi*-positive**	Positive	Positive	Positive	Positive
32820	1.3	80	80	***T. cruzi*-positive**	Positive	Negative	Negative	Positive
33384	3.5	160	80	***T. cruzi*-positive**	Positive	Positive	Positive	Positive
33748	3.3	320	80	***T. cruzi*-positive**	Positive	Positive	Positive	Positive
35084	4	160	80	***T. cruzi*-positive**	Positive	Positive	Positive	Positive

7022	2.5	320	160	***T. cruzi*-positive**	Positive	Positive	Positive	Positive
9097	2.6	1280	160	***T. cruzi*-positive**	Positive	Positive	Positive	Positive
9695	1.8	320	160	***T. cruzi*-positive**	Positive	Positive	Negative	Positive
9736	2.7	320	160	***T. cruzi*-positive**	Positive	Positive	Positive	Positive
10015	2.2	160	160	***T. cruzi*-positive**	Positive	Positive	Positive	Positive
10512	1.3	160	160	***T. cruzi*-positive**	Positive	Positive	Positive	Positive
10893	2	320	160	***T. cruzi*-positive**	Positive	Positive	Positive	Positive
10948	1.6	320	160	***T. cruzi*-positive**	Positive	Positive	Positive	Positive
11006	1.3	640	160	***T. cruzi*-positive**	Positive	Positive	Positive	Positive
12708	3.3	320	160	***T. cruzi*-positive**	Positive	Positive	Positive	Positive
12711	3.3	320	160	***T. cruzi*-positive**	Positive	Positive	Positive	Positive
13128	2.3	160	160	***T. cruzi*-positive**	Positive	Positive	Positive	Positive
13268	1.9	160	160	***T. cruzi*-positive**	Positive	Positive	Positive	Positive
13684	3.6	320	160	***T. cruzi*-positive**	Positive	Positive	Positive	Positive
13749	3.1	160	160	***T. cruzi*-positive**	Positive	Positive	Positive	Positive
14046	2	320	160	***T. cruzi*-positive**	Positive	Positive	Negative	Positive
14772	1.9	320	160	***T. cruzi*-positive**	Positive	Positive	Positive	Positive
15387	2.1	160	160	***T. cruzi*-positive**	Positive	Positive	Positive	Positive
16733	2	160	160	***T. cruzi*-positive**	Positive	Positive	Positive	Positive
17013	2.1	320	160	***T. cruzi*-positive**	Positive	Positive	Positive	Positive
17678	2	640	160	***T. cruzi*-positive**	Positive	Positive	Positive	Positive
18795	2.4	320	160	***T. cruzi*-positive**	Negative	Positive	Positive	Positive
19263	3	320	160	***T. cruzi*-positive**	Positive	Positive	Positive	Positive
19298	2.1	320	160	***T. cruzi*-positive**	Positive	Positive	Positive	Positive
19882	1.7	320	160	***T. cruzi*-positive**	Positive	Positive	Positive	Positive
20161	3.2	320	160	***T. cruzi*-positive**	Negative	Positive	Positive	Positive
26150	4.2	160	160	***T. cruzi*-positive**	Positive	Positive	Positive	Positive
26505	2.3	320	160	***T. cruzi*-positive**	Positive	Positive	Positive	Positive
27421	1.6	160	160	***T. cruzi*-positive**	Positive	Positive	Positive	Positive

29560	1.8	320	160	***T. cruzi*-positive**	Positive	Positive	Positive	Positive
29903	2.3	640	160	***T. cruzi*-positive**	Positive	Positive	Positive	Positive
30340	1.3	160	160	***T. cruzi*-positive**	Negative	Positive	Positive	Positive
32855	2.5	320	160	***T. cruzi*-positive**	Positive	Positive	Positive	Positive
33645	1.8	2560	160	***T. cruzi*-positive**	Positive	Positive	Positive	Positive
33791	2.5	640	160	***T. cruzi*-positive**	Negative	Positive	Positive	Positive
33828	1.8	5120	160	***T. cruzi*-positive**	Positive	Positive	Positive	Positive
34022	2.1	160	160	***T. cruzi*-positive**	Positive	Positive	Positive	Positive
34130	2.2	640	160	***T. cruzi*-positive**	Positive	Positive	Positive	Positive
34178	3.8	640	160	***T. cruzi*-positive**	Positive	Positive	Positive	Positive
34665	1.4	2560	160	***T. cruzi*-positive**	Positive	Positive	Positive	Positive
8762	1.3	160	320	***T. cruzi*-positive**	Positive	Positive	Positive	Positive
9143	2.5	320	320	***T. cruzi*-positive**	Positive	Positive	Positive	Positive
9212	2.3	160	320	***T. cruzi*-positive**	Positive	Positive	Positive	Positive
10889	1.5	160	320	***T. cruzi*-positive**	Positive	Positive	Positive	Positive
12246	1.4	320	320	***T. cruzi*-positive**	Positive	Positive	Positive	Positive
12560	2.7	160	320	***T. cruzi*-positive**	Positive	Positive	Positive	Positive
12723	2.9	160	320	***T. cruzi*-positive**	Positive	Positive	Positive	Positive
13995	2.2	320	320	***T. cruzi*-positive**	Positive	Positive	Positive	Positive
14006	2.6	160	320	***T. cruzi*-positive**	Positive	Positive	Positive	Positive
14237	2.8	160	320	***T. cruzi*-positive**	Positive	Positive	Positive	Positive
14370	2.6	160	320	***T. cruzi*-positive**	Positive	Positive	Positive	Positive
15064	3.2	160	320	***T. cruzi*-positive**	Positive	Positive	Positive	Positive
15112	4.6	320	320	***T. cruzi*-positive**	Positive	Positive	Positive	Positive
16132	2.3	160	320	***T. cruzi*-positive**	Positive	Positive	Positive	Positive
17075	2.9	320	320	***T. cruzi*-positive**	Positive	Positive	Positive	Positive
17174	1.8	640	320	***T. cruzi*-positive**	Positive	Positive	Positive	Positive
17687	1.7	160	320	***T. cruzi*-positive**	Positive	Positive	Positive	Positive
18131	2.8	640	320	***T. cruzi*-positive**	Positive	Positive	Positive	Positive

18372	4.5	1280	320	***T. cruzi*-positive**	Positive	Positive	Positive	Positive
21028	2.1	1280	320	***T. cruzi*-positive**	Positive	Positive	Positive	Positive
21141	1.5	640	320	***T. cruzi*-positive**	Negative	Positive	Positive	Positive
22550	2.7	1280	320	***T. cruzi*-positive**	Positive	Positive	Positive	Positive
26340	1.6	160	320	***T. cruzi*-positive**	Negative	Negative	Negative	Positive
27916	4.1	1280	320	***T. cruzi*-positive**	Positive	Positive	Positive	Positive
28273	1.9	160	320	***T. cruzi*-positive**	Positive	Positive	Positive	Positive
29232	2.6	160	320	***T. cruzi*-positive**	Positive	Positive	Positive	Positive
29357	1.5	160	320	***T. cruzi*-positive**	Positive	Positive	Positive	Positive
32654	2.7	320	320	***T. cruzi*-positive**	Positive	Negative	Positive	Positive
33601	1.5	160	320	***T. cruzi*-positive**	Positive	Positive	Positive	Positive
33621	1.5	640	320	***T. cruzi*-positive**	Positive	Positive	Positive	Positive
34365	2.3	160	320	***T. cruzi*-positive**	Positive	Positive	Positive	Positive
35535	3.7	80	320	***T. cruzi*-positive**	Positive	Positive	Positive	Positive
35716	1.6	320	320	***T. cruzi*-positive**	Positive	Positive	Positive	Positive
10609	1.9	160	640	***T. cruzi*-positive**	Positive	Positive	Positive	Positive
11609	1.6	160	640	***T. cruzi*-positive**	Positive	Positive	Positive	Positive
12648	3.1	640	640	***T. cruzi*-positive**	Positive	Positive	Positive	Positive
13053	1.6	1280	640	***T. cruzi*-positive**	Positive	Positive	Positive	Positive
13558	1.8	160	640	***T. cruzi*-positive**	Positive	Positive	Positive	Positive
13673	2.4	160	640	***T. cruzi*-positive**	Positive	Positive	Positive	Positive
14443	1.3	160	640	***T. cruzi*-positive**	Negative	Positive	Positive	Positive
15610	2.9	160	640	***T. cruzi*-positive**	Negative	Positive	Positive	Positive
17398	3.2	1280	640	***T. cruzi*-positive**	Positive	Positive	Positive	Positive
17542	2.9	1280	640	***T. cruzi*-positive**	Positive	Positive	Positive	Positive
19065	3.5	1280	640	***T. cruzi*-positive**	Positive	Positive	Positive	Positive
19996	4.2	320	640	***T. cruzi*-positive**	Positive	Positive	Positive	Positive
25885	2.4	640	640	***T. cruzi*-positive**	Positive	Positive	Positive	Positive
29042	3.3	320	640	***T. cruzi*-positive**	Positive	Positive	Positive	Positive

33675	2.6	5120	640	***T. cruzi*-positive**	Positive	Positive	Positive	Positive
33876	1.9	640	640	***T. cruzi*-positive**	Positive	Positive	Positive	Positive
11306	1.5	160	1280	***T. cruzi*-positive**	Positive	Positive	Positive	Positive
14413	3	160	1280	***T. cruzi*-positive**	Positive	Positive	Positive	Positive
18955	1.7	320	1280	***T. cruzi*-positive**	Positive	Positive	Positive	Positive
24925	1.9	1280	1280	***T. cruzi*-positive**	Positive	Positive	Positive	Positive
33627	2.3	5120	1280	***T. cruzi*-positive**	Positive	Positive	Positive	Positive
21589	0.8	<10	40	***T. cruzi*-negative**	Negative	Negative	Negative	Negative
26139	0.1	<10	80	***T. cruzi*-negative**	Negative	Negative	Negative	Negative
28364	0.2	10	80	***T. cruzi*-negative**	Negative	Negative	Negative	Negative
14148	0.8	<10	80	***T. cruzi*-negative**	Positive	Negative	Positive	Positive
24246	0.3	<10	160	***T. cruzi*-negative**	Negative	Negative	Negative	Negative
24547	0.3	<10	160	***T. cruzi*-negative**	Negative	Negative	Negative	Negative
21813	0.4	<10	320	***T. cruzi*-negative**	Negative	Negative	Negative	Negative
29845	0.4	<10	320	***T. cruzi*-negative**	Negative	Negative	Negative	Positive
9842	0.1	<10	640	***T. cruzi*-negative**	Negative	Negative	Negative	Negative
33690	0.5	<10	<40	***T. cruzi*-negative**	Negative	Negative	Negative	Negative
7438	0.2	<10	<40	***T. cruzi*-negative**	Negative	Negative	Negative	Negative
8998	0.2	<10	<40	***T. cruzi*-negative**	Negative	Negative	Negative	Negative
10558	0.7	<10	<40	***T. cruzi*-negative**	Negative	Negative	Negative	Negative
11589	0.6	<10	<40	***T. cruzi*-negative**	Negative	Negative	Negative	Negative
12162	0.6	10	<40	***T. cruzi*-negative**	Negative	Negative	Negative	Negative
15133	0.2	20	<40	***T. cruzi*-negative**	Negative	Negative	Negative	Negative
15452	0.4	<10	<40	***T. cruzi*-negative**	Negative	Negative	Negative	Positive
15898	0.3	80	<40	***T. cruzi*-negative**	Negative	Negative	Negative	Negative
16952	0.4	10	<40	***T. cruzi*-negative**	Negative	Negative	Negative	Negative
17651	0.5	<10	<40	***T. cruzi*-negative**	Negative	Negative	Negative	Negative
19739	0.7	80	<40	***T. cruzi*-negative**	Positive	Negative	Negative	Positive
20688	0.2	20	<40	***T. cruzi*-negative**	Negative	Negative	Negative	Negative

22250	0.2	<10	<40	***T. cruzi*-negative**	Negative	Negative	Negative	Negative
24487	0.2	80	<40	***T. cruzi*-negative**	Negative	Negative	Negative	Negative
27400	0.2	160	<40	***T. cruzi*-negative**	Negative	Negative	Negative	Negative
27692	0.4	40	<40	***T. cruzi*-negative**	Negative	Negative	Negative	Negative
27693	0.1	40	<40	***T. cruzi*-negative**	Negative	Negative	Negative	Positive
30528	0.4	40	<40	***T. cruzi*-negative**	Negative	Negative	Negative	Negative
33606	0.3	<10	<40	***T. cruzi*-negative**	Negative	Negative	Negative	Negative
33711	0.2	10	<40	***T. cruzi*-negative**	Negative	Negative	Negative	Negative
33735	0.1	<10	<40	***T. cruzi*-negative**	Negative	Negative	Negative	Negative
33741	0.2	<10	<40	***T. cruzi*-negative**	Negative	Negative	Negative	Negative
33799	0.2	10	<40	***T. cruzi*-negative**	Negative	Negative	Negative	Negative
33800	0.2	<10	<40	***T. cruzi*-negative**	Negative	Negative	Negative	Negative
33868	0.2	<10	<40	***T. cruzi*-negative**	Negative	Negative	Negative	Negative
33872	0.2	<10	<40	***T. cruzi*-negative**	Negative	Negative	Negative	Negative
34300	0.4	10	<40	***T. cruzi*-negative**	Negative	Negative	Negative	Negative
34319	0.2	<10	<40	***T. cruzi*-negative**	Negative	Negative	Negative	Negative
34415	0.6	<10	<40	***T. cruzi*-negative**	Negative	Negative	Negative	Positive
34423	0.6	10	<40	***T. cruzi*-negative**	Negative	Negative	Negative	Negative
34465	0.2	10	<40	***T. cruzi*-negative**	Negative	Negative	Negative	Negative
34478	0.3	<10	<40	***T. cruzi*-negative**	Negative	Negative	Negative	Negative
34484	0.4	<10	<40	***T. cruzi*-negative**	Negative	Negative	Negative	Negative
34527	0.4	<10	<40	***T. cruzi*-negative**	Negative	Negative	Negative	Negative
34795	0.3	10	<40	***T. cruzi*-negative**	Negative	Negative	Negative	Negative
34810	0.4	<10	<40	***T. cruzi*-negative**	Positive	Negative	Negative	Positive
34842	0.2	<10	<40	***T. cruzi*-negative**	Negative	Negative	Negative	Negative
34877	0.3	<10	<40	***T. cruzi*-negative**	Negative	Negative	Negative	Negative
34888	0.4	<10	<40	***T. cruzi*-negative**	Negative	Negative	Negative	Negative
34977	0.2	10	<40	***T. cruzi*-negative**	Negative	Negative	Negative	Negative
34983	0.3	10	<40	***T. cruzi*-negative**	Negative	Negative	Negative	Positive

35096	0.4	<10	<40	***T. cruzi*-negative**	Negative	Negative	Negative	Negative
35104	0.2	10	<40	***T. cruzi*-negative**	Positive	Negative	Negative	Negative
35133	0.5	<10	<40	***T. cruzi*-negative**	Positive	Negative	Negative	Negative
35167	0.3	<10	<40	***T. cruzi*-negative**	Negative	Negative	Negative	Negative
12347	0.5	10	<40	***T. cruzi*-negative**	Positive	Negative	Negative	Positive
21299	0.5	<10	<40	***T. cruzi*-negative**	Negative	Negative	Negative	Positive
29125	0.4	40	<40	***T. cruzi*-negative**	Positive	Negative	Negative	Positive
29545	0.5	<10	<40	***T. cruzi*-negative**	Positive	Negative	Negative	Positive
13401	0.5	40	<40	**Cutaneous leishmaniosis**	Negative	Positive	Positive	Negative
14668	0.6	320	<40	**Cutaneous leishmaniosis**	Negative	Negative	Negative	Negative
16183	0.7	160	80	**Cutaneous leishmaniosis**	Positive	Negative	Negative	Negative
25543	0.8	320	320	**Cutaneous leishmaniosis**	Negative	Negative	Negative	Negative
12830	0.2	20	80	**Cutaneous leishmaniosis**	Positive	Positive	Positive	Positive
15382	0.22	40	<40	**Cutaneous leishmaniosis**	Negative	Positive	Positive	Positive
30167	0.9	1280	<40	**Visceral leishmaniosis**	Negative	Negative	Negative	Negative
33803	0.1	2560	40	**Visceral leishmaniosis**	Negative	Negative	Negative	Negative
35804	0.12	40	<40	**Visceral leishmaniosis**	Negative	Negative	Negative	Negative
35805	0.13	160	320	**Visceral leishmaniosis**	Negative	Negative	Negative	Negative
35806	0.14	320	40	**Visceral leishmaniosis**	Negative	Negative	Negative	Negative
35808	0.16	320	40	**Visceral leishmaniosis**	Negative	Negative	Negative	Negative
1884	0.18	160	80	**Visceral leishmaniosis**	Negative	Positive	Positive	Negative
1969	0.19	2,560	320	**Visceral leishmaniosis**	Negative	Positive	Positive	Negative
14294	0.21	40	<40	**Visceral leishmaniosis**	Negative	Positive	Positive	Positive
32676	0.23	1,280	<40	**Visceral leishmaniosis**	Negative	Negative	Positive	Positive
35802	0.11	<10	40	**Toxoplasmosis**	Negative	Negative	Negative	Negative
35807	0.15	<10	<40	**Toxoplasmosis**	Negative	Negative	Negative	Positive
35809	0.17	10	<40	**Toxoplasmosis**	Negative	Negative	Negative	Negative
35803	0.24	40	<40	**Toxoplasmosis**	Negative	Negative	Negative	Negative
